# Preliminary exploration of survival analysis using the OHDSI common data model: a case study of intrahepatic cholangiocarcinoma

**DOI:** 10.1186/s12911-018-0686-7

**Published:** 2018-12-07

**Authors:** Na Hong, Ning Zhang, Huawei Wu, Shanshan Lu, Yue Yu, Li Hou, Yinying Lu, Hongfang Liu, Guoqian Jiang

**Affiliations:** 1 Department of Health Sciences Research, Mayo Clinic, Rochester, MN USA; 2 Integrated TCM & Western Medicine Department, 302 Military Hospital, Beijing, China; 3 Chengdu Library and Information Center, University of Chinese Academy of Sciences, Chengdu, China; 4 Comprehensive Liver Cancer Center, 302 Military Hospital, Beijing, China; 5 Institute of Medical Information, Chinese Academy of Medical Sciences, Beijing, China

**Keywords:** OHDSI CDM, Survival analysis, Multi-center analysis, R, Intrahepatic cholangiocarcinoma

## Abstract

**Background:**

Data heterogeneity is a common phenomenon related to the secondary use of electronic health records (EHR) data from different sources. The Observational Health Data Sciences and Informatics (OHDSI) Common Data Model (CDM) organizes healthcare data into standard data structures using concepts that are explicitly and formally specified through standard vocabularies, thereby facilitating large-scale analysis. The objective of this study is to design, develop, and evaluate generic survival analysis routines built using the OHDSI CDM.

**Methods:**

We used intrahepatic cholangiocarcinoma (ICC) patient data to implement CDM-based survival analysis methods. Our methods comprise the following modules: 1) Mapping local terms to standard OHDSI concepts. The analytical expression of variables and values related to demographic characteristics, medical history, smoking status, laboratory results, and tumor feature data. These data were mapped to standard OHDSI concepts through a manual analysis; 2) Loading patient data into the CDM using the concept mappings; 3) Developing an R interface that supports the portable survival analysis on top of OHDSI CDM, and comparing the CDM-based analysis results with those using traditional statistical analysis methods.

**Results:**

Our dataset contained 346 patients diagnosed with ICC. The collected clinical data contains 115 variables, of which 75 variables were mapped to the OHDSI concepts. These concepts mainly belong to four domains: condition, observation, measurement, and procedure. The corresponding standard concepts are scattered in six vocabularies: ICD10CM, ICD10PCS, SNOMED, LOINC, NDFRT, and READ. We loaded a total of 25,950 patient data records into the OHDSI CDM database. However, 40 variables failed to map to the OHDSI CDM as they mostly belong to imaging data and pathological data.

**Conclusions:**

Our study demonstrates that conducting survival analysis using the OHDSI CDM is feasible and can produce reusable analysis routines. However, challenges to be overcome include 1) semantic loss caused by inaccurate mapping and value normalization; 2) incomplete OHDSI vocabularies describing imaging data, pathological data, and modular data representation.

## Background

Survival analysis is a widely used statistical method for the retrospective study of clinical data. It is widely used in the field of medical research, especially for the observational study of tumors associated with definite events. Examples include exploring the probability of the occurrence and recurrence of tumors, and the probability of death and risk factors related to tumors. Specifically, it includes methods such as Kaplan-Meier estimation, life table analysis, Cox proportional hazards regression analysis, and the log-rank test. Survival analysis addresses the following research questions such as the probability of survival past a certain time, the rate of event occurrence at a certain time, and the risk factors that contribute to the event.

To acquire sufficient statistical evidence from clinical data and generate more reliable conclusions, observational medical research is often conducted across multi-center institutions [1]. This can balance the various biases present in single-center research, leading to higher-quality results. A number of multi-center prospective observational studies have been conducted [2–7], concluding that large-scale data analysis from multiple centers may provide more reliable conclusions.

However, data heterogeneity is a common phenomenon in the secondary use of Electronic Health Records (EHR) data collected from different sources. Inconsistent data formats hinder the multi-center analysis of clinical data. A lack of a common data infrastructure and consistent terminology precludes large-scale clinical research collaboration across institutes. Some researchers have shown that analyzing EHR data using standard-based methods is economical and efficient [8]. By standardizing the data structures from different institutions, it is possible to combine data from different research centers for analysis, and allow the use of raw EHR data to improve efficiency.

A number of Common Data Model (CDM)-based research initiatives, including the National Patient-Centered Research Networks (PCORnet) (http://www.pcornet.org/resource-center/pcornet-common-data-model/), the Informatics for Integrating Biology and the Bedside (i2b2) (https://www.i2b2.org/), and the Observational Health Data Sciences and Informatics (OHDSI) (https://www.ohdsi.org/) [9], have been launched to take efforts towards enabling standards-based clinical data research [10–12].

Among them, OHDSI is a worldwide non-profit research alliance that focuses on open-source solutions for medical big data analysis. The OHDSI CDM provides standard-based data analysis solutions that support converting EHR data from different sources into a standard data structure, representing EHR data using semantically-consistent concepts, and conducting large-scale data analysis. To support the implementation, OHDSI provides a set of tools for data conversion, analysis, and visualization [9, 13]. A large-scale multi-center survival analysis conducted using the OHDSI platform facilitates an effective clinical decision-making system based on real-world clinical data. This alleviates some of the current shortcomings in disease diagnosis and prognosis research.

We use intrahepatic cholangiocarcinoma (ICC) patient data to support our case study. ICC is a type of highly heterogeneous malignant tumor. Studies have shown that the incidence of ICC is on the rise worldwide, and the mortality rate is also increasing. To improve the overall treatment status and prognosis of ICC, it is necessary to conduct a systematic synthesis of large-scale real-world research data.

In this study, we collected real ICC patient EHR data to evaluate the feasibility of building survival analysis models using the OHDSI CDM. We developed generic survival analysis routines that are based on the OHDSI CDM standards for portable analysis and future rapid reuse on EHR data from multiple sources of EHR data.

## Methods

### Data

#### Patient cohort selection

We included patients who were newly diagnosed with ICC histopathologically, and treated at Beijing 302 Hospital between July 2007 and July 2017. Patients were excluded using criteria as follows: hilar or distal ICC, intrahepatic metastasis of extrahepatic cholangiocarcinoma (ECC), mixed with hepatocellular carcinoma (HCC), uncertain origin or benign mass, perioperative mortality (defined as 1 month after operation), and combinations with other malignancies. Patients with only once medical record and loss of follow-up thereafter or those with incomplete information were also excluded.

#### Data collection

We collected the following clinical data on the ICC patients: demographic characteristics, medical history, smoking status, laboratory results, and tumor features. All clinical data were abstracted at the time of diagnosis prior to specific anti-cancer therapy. Patients were followed up for death or recurrence of ICC from the time of diagnosis to July 31, 2017.

#### The OHDSI CDM

The OHDSI CDM is designed to include all observational health data elements to support the generation of reliable scientific evidence. It is essentially a relational database representing observational data derived from the EHR. In the OHDSI CDM data tables, the meaning of each portion of content is represented using standard concepts. Content-related concepts are stored with their concept_ids as foreign keys to the CONCEPT table in the standardized vocabularies. We used the V5.3 OHDSI CDM (https://github.com/OHDSI/CommonDataModel) for our local table schemas. It contains 37 tables and 42 vocabularies.

#### R packages

We used two R packages in our experiments. The first one is the survival package (https://cran.r-project.org/web/packages/survival/index.html), which was used to support core survival analysis, including the definition of survival objects, the Kaplan-Meier estimation, and the Cox model analysis. The second one is the R Mice package (https://cran.r-project.org/web/packages/mice/index.html). Missing data is a ubiquitous problem with clinical research data. For example, blood pressure measurements may be missing because of the breakdown of an automatic sphygmomanometer. Many current analysis tools can only handle a complete data sets, thus, we use the R Mice package to impute missing values.

### Overall method framework

**Fig. 1:**
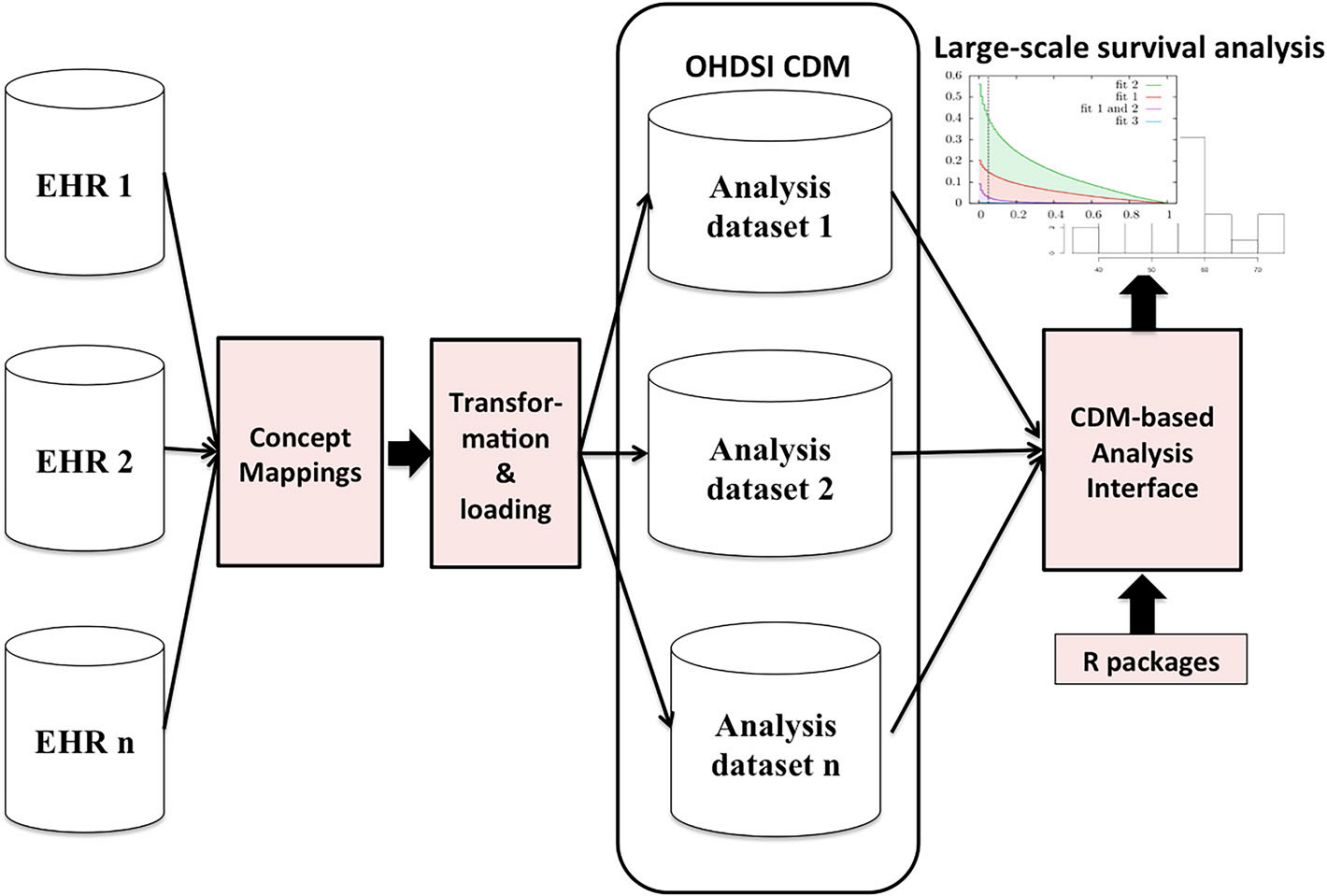
The OHDSI-CDM-based generic survival analysis framework

We designed an OHDSI CDM-supported survival analysis framework to facilitate large-scale multi-center survival analysis, as shown in Fig. 1. The framework contains the following three key modules: mapping local terms to standard concepts, Extraction-Transformation-Loading (ETL) of patient data into OHDSI CDM, and developing a generic analysis interface with the OHDSI CDM.

#### Mapping local terms to standard concepts

We analyzed all of the tables and vocabularies of the OHDSI CDM and manually created mappings from the analytical variables in the patient data to the corresponding CDM tables and concepts and normalized the value expressions. The collected ICC patient data was used as the source EHR data for the mapping study.

Six main categories of ICC patient data are used for survival analysis. 1) Demographic characteristics, including date of birth, gender, country, ethnicity, and blood type. 2) Medical history including comorbidities prior to ICC diagnosis (e.g., diabetes, hypertension, cholelithiasis, hyperlipidemia, coronary artery disease, and cholecystectomy). Underlying liver disease was either abstracted and confirmed by reviewing the medical records or ascertained through related clinical observations. 3) Laboratory results with parameters involving, among others, leukocytes, erythrocytes, platelets, albumin, total bilirubin, creatinine, carbohydrate antigen (CA) 19–9, and CA 125. 4) Smoking status was manually abstracted from the admission medical records of patients. 5) Therapeutic procedures conducted on each patient. 6) Tumor features such as tumor number, maximum size, vascular invasion, and lymph node involvement were assessed using contrast computed tomography (CT), magnetic resonance imaging (MRI) examination, or pathological reports of the patients.

**Fig. 2:**
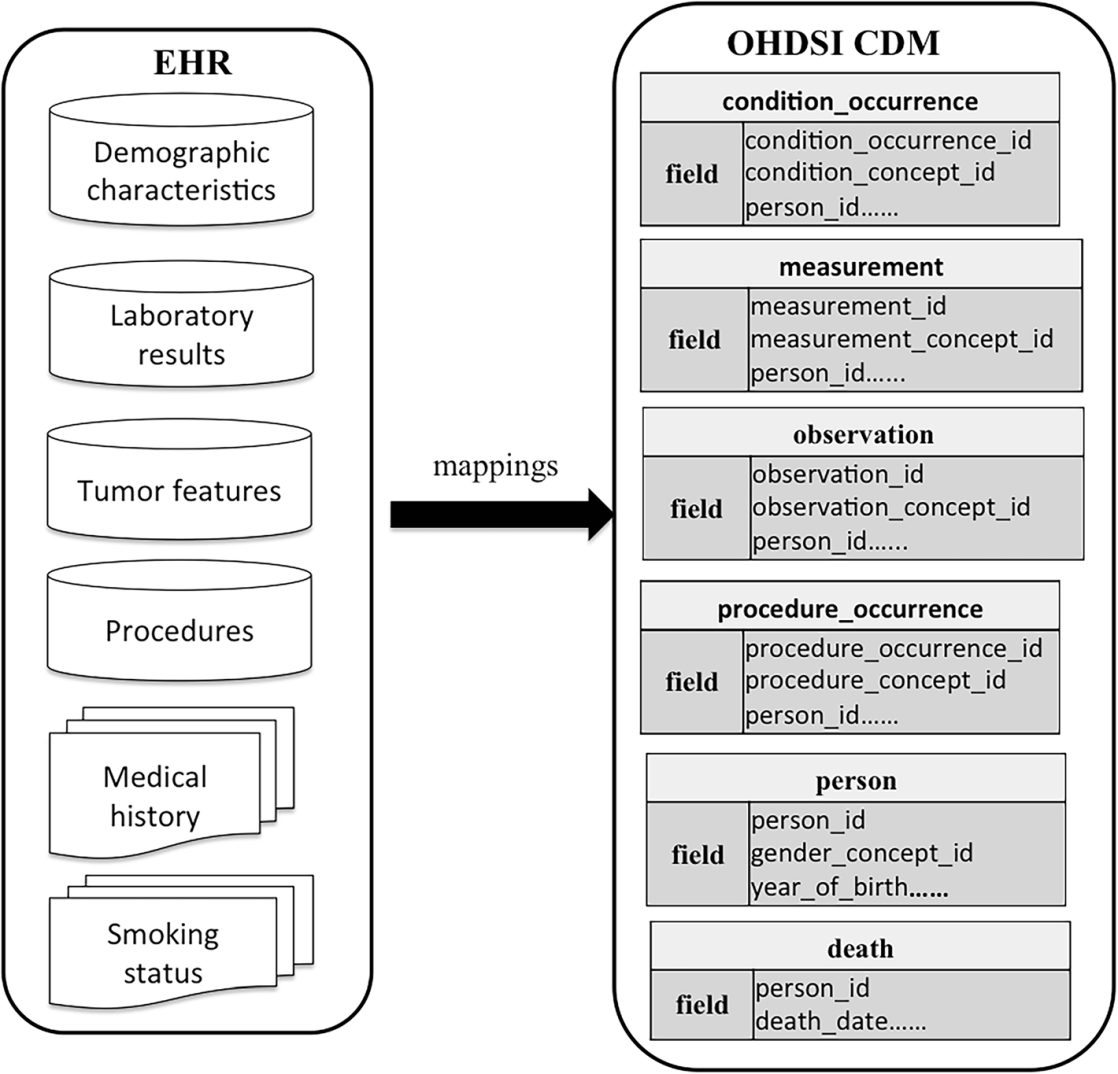
Mappings between source EHR terms and standard OHDSI concepts

Creating mappings between the source variables and their values to the target OHDSI concepts is crucial to facilitate patient data standardization. Because the source variable names are typically expressed in non-standard terms, and the textual variable values are often in free-style using different local expressions, we must standardize these terms and the textual values into standard concepts. The mappings between the source EHR data terms and the OHDSI CDM concepts were created as shown in Fig. 2. We used the OHDSI vocabulary browser Athena (http://athena.ohdsi.org/) to help find the corresponding standard concept in OHDSI when conducting concept mappings. To ensure minimal mapping errors and minimal information loss, two authors reviewed the concept mappings to achieve agreement.

#### Transformation and loading of patient data into the OHDSI CDM

To load patient data into the OHDSI CDM, we developed a data transformation and loading algorithm to populate the ICC patient data into the CDM using the above concept mappings. There are three key steps for patient data transformation and loading. 1) The variable grouping. We manually categorized the source variables with respect to the target OHDSI tables. Six core tables are involved in our data loading: condition_occurrence [medical history], measurement [laboratory results], observation [tumor features and smoking status], procedure_occurrence [procedures], person [demographic characteristics], and death [vital status] Table. 2) De-identification. To maintain patient confidentiality privacy and security, when the patient data were loaded into the OHDSI CDM, the original patient_ids were removed and the CDM generated random person_ids for OHDSI research data management and analysis purposes. 3) Missing data imputation. The R package Mice provides the general approaches to deal with missing data for multivariate scenarios [14]. The Mice package currently offers more than 20 different methods to for different situations. The “random forest” imputation is one of the most commonly used methods in the Mice framework, and [15] recommends this method for imputing complex research data sets. Therefore, we used the function mice (data, method = “rf”) to impute our missing ICC patient data. 4) Loading patient data into the CDM. According to the concept mappings, we developed a set of transformation scripts to directly load the patient data into the corresponding six core tables. The associated relationships with other indirect tables, such as concepts, vocabulary, and domain, were generated through “concept_id”.

#### Developing a generic analysis interface in the OHDSI CDM

Once the patient data are loaded into the OHDSI CDM, the scalable survival analysis across multiple sites is facilitated. We developed a generic survival analysis interface in the OHDSI CDM to enable the reusable R functions using the R ‘survival’ package. The event of interest is patient death and the overall survival (OS) is defined as the interval between the diagnosis and death or the date of the last contact with the subjects. Our R interface supports the general survival analysis functions in the CDM, and currently includes: 1) Creating the analysis dataset which relies on a set of input variants ‘concept_id’ because ‘concept_id’ is the standard identifier of each analysis object. Furthermore, ‘concept_id’ is independent with respect to platforms, and offers extensible interoperability with other OHDSI CDM applications, such as cohort query results. 2) Constructing the baseline demographics study that includes predefined analysis functions such as frequency, percentage, mean ± standard deviation, and the interquartile range (IQR). 3) Building the Kaplan-Meier survival curve, defined as the probability of surviving within a given length of time, considering time in many small intervals [16].

The CDM-based R analysis tool runs in the local environment within a single institute to ensure the security of the personal health identification (PHI) information. Our methods also support the capability portable among multiple institutes that deploy the OHDSI CDM.

#### Evaluation of the CDM-based results

To evaluate the CDM-based analysis results, we performed the analysis using the source ICC patient data with the common analysis tool Intercooled Stata 13.0 (https://www.stata.com/), and compared the results for a group of specific analysis tasks.

## Results

In total, we used data from 346 ICC patients for survival analysis. The data set contains data specified in 115 variables of which 75 were mapped to the OHDSI concepts. Except for the patient demographics, most of the target concepts belong to the following four domains: condition, observation, measurement, and procedure. In addition, variable concepts were standardized using six vocabularies: ICD10CM (https://www.cdc.gov/nchs/icd/icd10cm.htm), ICD10PCS (https://www.cms.gov/Medicare/Coding/ICD10/2018-ICD-10-PCS-and-GEMs.html), SNOMED (https://www.snomed.org/snomed-ct), LOINC (https://loinc.org/), NDFRT (http://bioportal.bioontology.org/ontologies/NDFRT?p=classes&conceptid=root), and READ (https://digital.nhs.uk/services/terminology-and-classifications/read-codes), as shown in Table 1. According to the manually created mappings, a total of 25,950 records of patient data were loaded into the OHDSI database, as shown in Table 2.

**Table 1:** The concept-standardized process in OHDSI (Measurement and Observation as examples)

Variable name from EHR	OHDSI_concept_id	Domain	Vocabulary	CODE	Standard name in OHDSI
RBC	3,035,569	Measurement	LOINC	5171–4	Red Blood Cell/erythrocyte
HGB	3,035,970	Measurement	LOINC	2283–0	Hemoglobin Denver [Presence] in Blood
CA125	45,422,082	Observation	READ	44a6.00	CA123_level
BMI	4,245,997	Observation	SNOMED	60,621,009	Body mass index

**Table 2:** The variable mapping analysis results

CDM Tables	Number of mapped variables	Number of loaded instances	Number of variables failed to be mapped	Example of variable that failed to be mapped
Condition_occurrence	16	5536	0	
Observation	20	6920	11	pathdiameterG, margininvasion, CTPgrade
Measurement	34	11,764	29	DBILRATIO, apA1, CA199.G, AFPL3.AFP
Procedure_occurrence	1	346	0	
Person	3	1038	0	
Death	1	346	0	
Total	75	25,950	40	

We implemented a generic analysis interface in the CDM by initiating an analysis dataset using OHDSI concepts. An example dataset is: SA_dataset = [cohort, ‘4146792’, ‘40482950’, variable_concept_IDs, site_ID], where cohort is the identified cohort, ‘4146792’ is the standard concept ‘Status’, and ‘40482950’ is the standard concept ‘Survival time’. All of the variable_concept_IDs are the target analytic variables represented as standard OHDSI concepts, and site_ID represents different OHDSI servers distributed across multiple institutes.

**Fig. 3:**
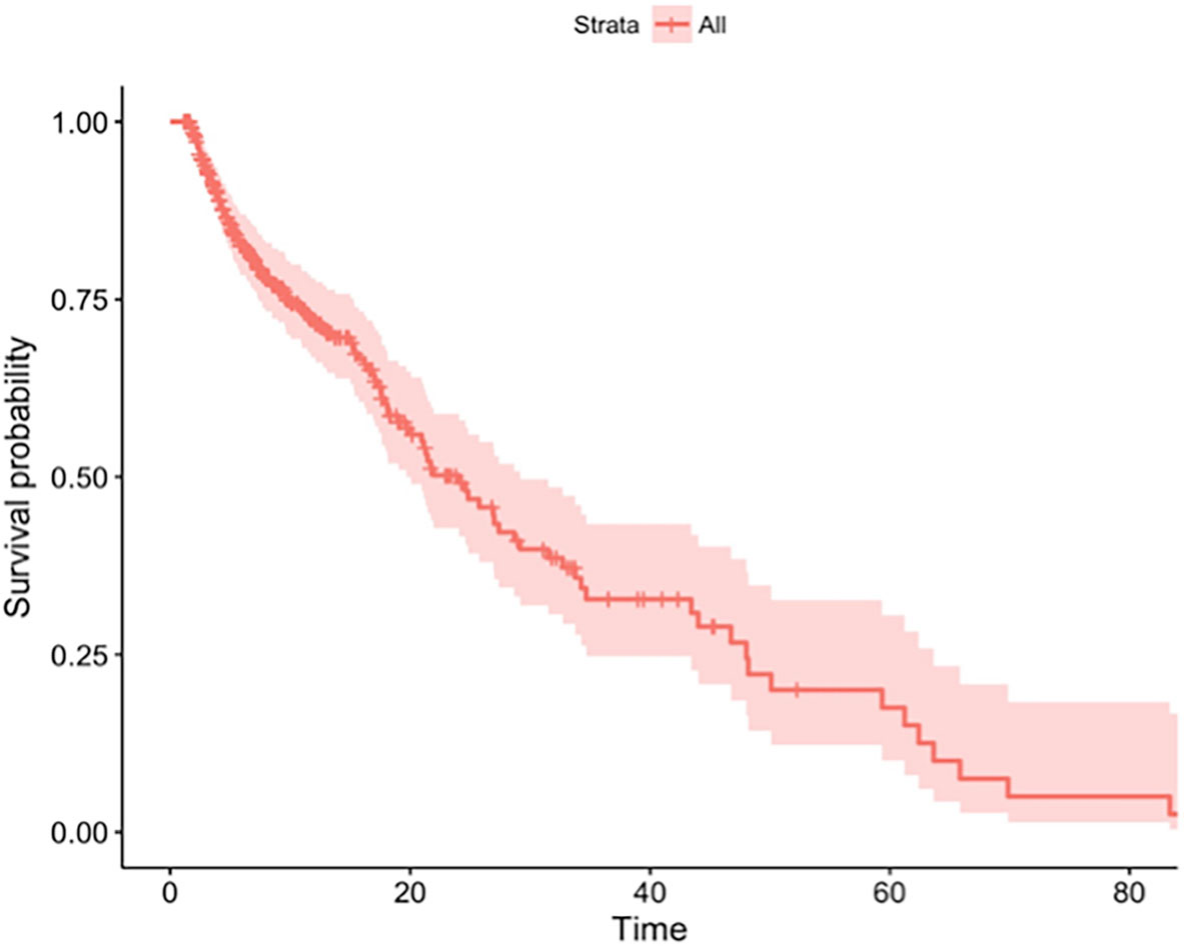
Kaplan-Meier survival curves for ICC patients by all variables

**Fig. 4:**
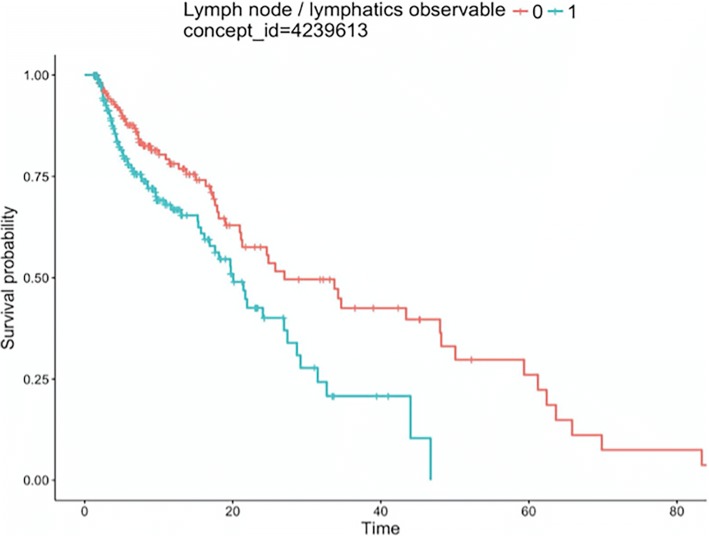
Kaplan-Meier survival curves for ICC patients by Lymph node / lymphatics observable

**Fig. 5:**
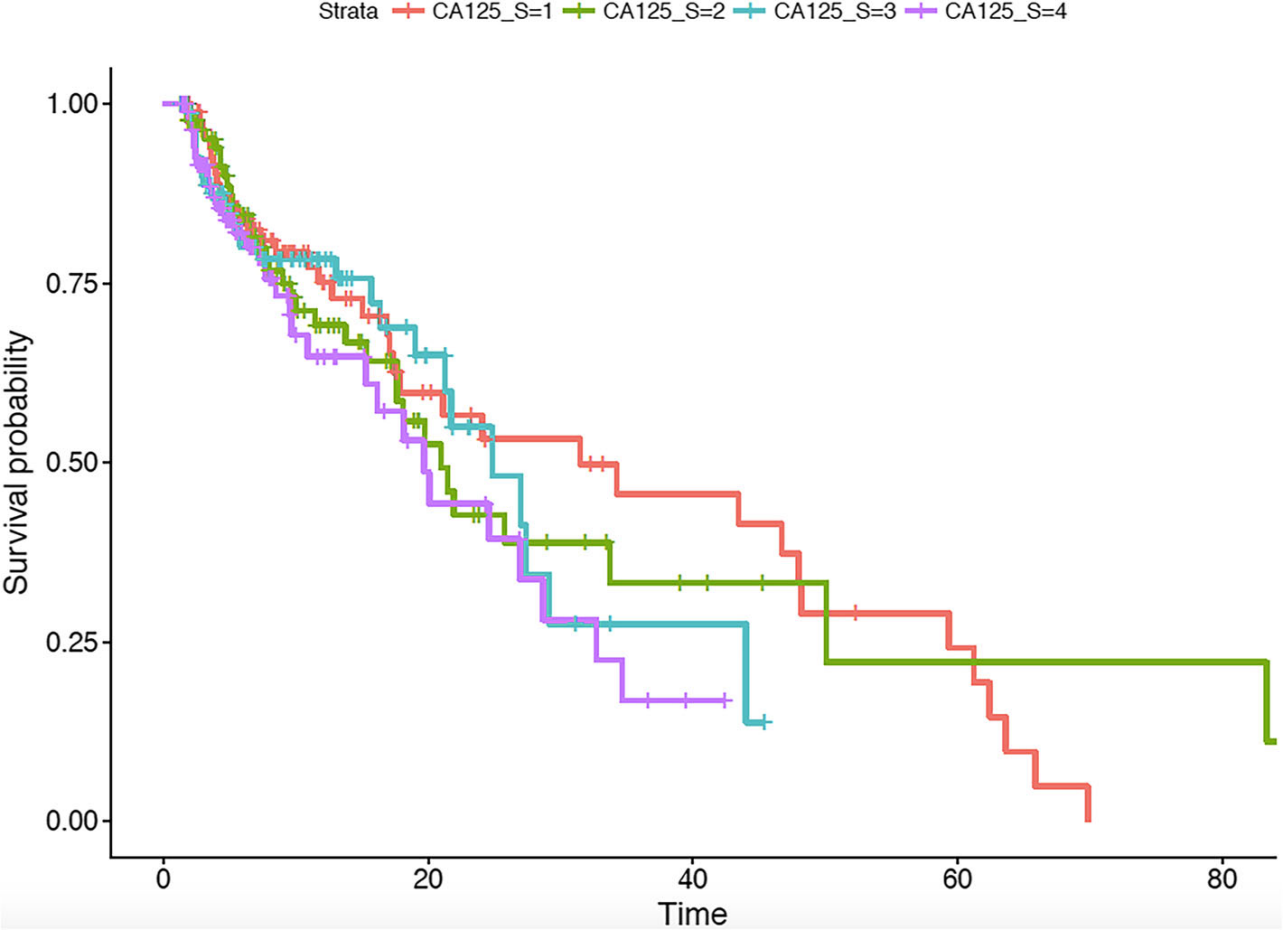
Kaplan-Meier survival curves for ICC patients by CA125 level

We then tested the CDM-based interface by generating summary statistics with respect to patient demographics and tumor characteristics and depicted the survival curves using the Kaplan-Meier method. We used the log-rank test to compare the survival curves of the ICC patients. Table 3 shows the demographics of the study population, from the table we conclude that 69.1% of the patients were male; One hundred and ninety-six patients had cirrhosis of liver; Lymph node status was available for 184 patients (53.2%), and the interquartile range of CA125 level is 24.07 (12.55–71.96). Additionally, Fig. 3, Fig. 4 and Fig. 5 show examples of the survival curves according to all variables, variable Lymph node / lymphatics observable, and variable CA125 level, respectively. For evaluation purposes, we compared the analysis results with respect to variables that mapped to the OHDSI with the Stata-based results. The CDM-based results were consistent with the Stata-based results on the same analysis variables. However, the entire analysis may not be fully supported by CDM because a number of variables (40 out of 115 in our use case) failed to be mapped to the OHDSI concepts.

**Table 3:** Demographics of study population

concept_id: 4135376: Gender (%)	69.1/30.9
concept_id: 4064161: Cirrhosis of liver (%)	196 (56.6)
concept_id: 4239613: Lymph node / lymphatics observable (%)	184 (53.2)
concept_id: 45422082: CA125 level (IQR)	24.07 (12.55–71.96)

## Discussion

With the implementation of concept mappings, the ETL process, and generic R interfaces, we conducted a preliminary study on incorporating the OHDSI CDM and R tools to support survival analysis on real ICC patient data. In general, we demonstrate that our approach is effective in supporting survival analysis using the OHDSI CDM and can produce reusable and portable analysis routines.

However, we encountered some challenges during our study. 1) Imaging data and molecular data are not adequately handled by the OHDSI vocabularies. We recommend that the OHDSI community enhance the OHDSI CDM to support pathology and biomarker data representation, so as to support more comprehensive data analysis. 2) We observed inaccurate mapping issues. For example, there is a concept “Alanine aminotransferase/Aspartate aminotransferase [Enzymatic activity ratio] in Serum or Plasma” (concept_id = 3,019,056) in OHDSI, but EHR data may have separate laboratory test results for Alanine aminotransferase (ALT) and Aspartate aminotransferase (AST). In this case, when we load data into OHDSI, an additional ratio calculation is required. Semantic loss may exist between the EHR term and the standard OHDSI concept, caused by partial semantic matching or semantic ambiguity. 3) There is a value set normalization issue. During our data transformation, we noticed that there are different value expressions for different variables, such as the value of the concept ‘gender’ (concept_id = 4,135,376), whereby the values ‘M’ and ‘F’ must be transformed into standard concepts Male (concept_id = 8507) and Female (concept_id = 8530). Because the value expression is quite diverse, handling value set standardization requires additional methods and resources. In this study, we have not fully solved this issue.

The limitations of our work are as follows. First, we conducted our experiments using data from only one institution. We will conduct subsequent research to use multi-center data for large-scale survival analysis and further validate our methods. Second, we have not yet used the patient cohort identification interface in our methods. We will examine the interoperability of our analysis interface with the existing OHDSI cohort definition tools in our further study.

## Conclusion

In this study, we perform ICC patient survival analysis as a use case to explore a generic analysis framework using the OHDSI CDM. We mapped the raw EHR data to the OHDSI concepts, and unified data representation using the OHDSI CDM and standard vocabularies. In addition, we developed an R-based generic survival analysis interface to enable portability. The designed framework provides generic and scalable analysis capabilities that are applicable not only to ICC patient data analysis, but also to EHR data analysis for other diseases.

## Abbreviations

ALT: Alanine aminotransferase
AST: Aspartate aminotransferase
CDM: Common Data Model
CT: Computed Tomography
ECC: Extrahepatic Cholangiocarcinoma
EHR: Electronic Health Records
HCC: Hepatocellular Carcinoma
ICC: Intrahepatic Cholangiocarcinoma
ICD10CM: International Classification of Diseases, Tenth Revision, Clinical Modification
ICD10PCS: International Classification of Diseases, Tenth Revision, Procedure Coding System
IQR: Interquartile Range
LOINC: Logical Observations Identifiers, Names, Codes
MRI: Magnetic Resonance Imaging
NDFRT: The National Drug File - Reference Terminology
OHDSI: The Observational Health Data Sciences and Informatics
OS: Overall Survival
PHI: Personal Health Identification
SNOMED: Systematized Nomenclature of Medicine
